# Trustworthiness criteria for meta‐analyses of randomized controlled studies: OBGYN journal guidelines

**DOI:** 10.1002/uog.29118

**Published:** 2024-10-02

**Authors:** The OBGYN Editors' Integrity Group (OGEIG), Jason Abbott, Ganesh Acharya, Amir Aviram, Kurt Barnhart, Vincenzo Berghella, Catherine S. Bradley, Nancy Chescheir, Thomas D'Hooghe, Michael Geary, Janesh Gupta, Karl Oliver Kagan, Anthony Odibo, Aris Papageorghiou, Luis Sanchez‐Ramos, Donna Santillan, Elizabeth Stringer, Togas Tulandi, Susan C. Modesitt

## INTRODUCTION

Editors of a number of OBGYN journals have formed an OBGYN Editors' Integrity Group (OGEIG), with the aim of collaborating to improve trustworthiness in published papers^1^. These Editors have already jointly agreed and published quality criteria for randomized controlled trials (RCTs), incorporating the guidance in their instructions to authors^2^. The requirements for RCTs include:

1. prospective registration in an international clinical trials registry platform, with registration number provided;
2. statement of ethical approval, with name of approving committee;
3. statement of informed consent;
4. statement of adherence to CONSORT guidelines (and, ideally, provision of a CONSORT checklist to be published as supplementary material);
5. and a data‐sharing statement^2^.

Meta‐analysis is a quantitative statistical technique used to combine and analyze data from the results of multiple previous independent studies on a particular topic, to derive overall conclusions or effect estimates. In general (but not exclusively), meta‐analyses are based on RCTs. The results are often used to develop standard practice or clinical guidelines. However, RCTs may be inaccurate or fabricated, leading to journal withdrawal or retraction. This article aims to expand upon the list of RCT quality criteria for authors of meta‐analyses of RCTs, so that low‐quality and fabricated studies are excluded from meta‐analyses.

## METHODS

The Editors in the group were invited to participate in monthly or bimonthly calls regarding trustworthiness in OBGYN publishing, with the aim of preventing publication of untrustworthy science in women's health. Using data from the published literature, including our prior work^1^, ^2^, Cochrane guidance^3^, the TRACT checklist^4^, the author instructions of the various journals, and other publications related to trustworthiness of meta‐analyses of RCTs^5^, criteria for meta‐analyses were reviewed, reaching consensus by majority.

## RESULTS

By consensus, 21 quality criteria were agreed upon by the Editors. The aim is for authors to check and confirm the quality criteria for *each* identified RCT when carrying out a meta‐analysis of RCTs (Tables 1 and 2). These criteria help to identify trustworthy RCTs and are assigned to two groups: ‘absolute’ criteria and ‘other quality’ criteria. ‘Absolute’ trustworthiness criteria are those that, if not met, would suggest non‐inclusion in the main results of meta‐analyses of RCTs (Table 1). ‘Other quality’ criteria are those that, if not met, would suggest lower quality of RCTs (Table 2). In addition, the meta‐analysis should be prospectively registered in the PROSPERO database (or a similar international, publicly accessible database, e.g. INPLASY; Research Registry – Registry of Systematic Review/Meta‐Analysis).

**Table 1:** ‘Absolute’ RCT trustworthiness criteria

Domain/Item	Statement	Checked?
Governance		
Retraction	The RCT has not been retracted *(Check PubMed* ^7^ *and RetractionWatch* ^8^ *)*	_____
Registration	The RCT was preregistered in a publicly available international clinical trials registry prior to randomization *(for RCTs commencing ≥ 2010)*	_____
Ethics	The RCT was approved by an ethics committee or an institutional review board (IRB)	_____
Consort	Statement that CONSORT guidance^9^ was followed *(for RCTs commencing ≥ 2010)*	_____
	In addition, it is preferred but not mandatory that the RCT includes: CONSORT algorithm/diagram	_____
	CONSORT checklist (25 items)	_____
Outcome		
Outcome variation	The primary outcome of the RCT is consistent with the stated primary outcome in the RCT registration	_____

In general, if an article does not meet all criteria, this would suggest non‐inclusion in the main results of a meta‐analysis of RCTs.

**Table 2:** ‘Other quality’ RCT criteria*

Domain/Item	Statement	Checked?
Governance		
Retraction	The article does not have an expression of concern (EoC) attached *(Check PubMed* ^7^ *and RetractionWatch* ^8^ *)*	_____
Registration	RCT registration details are included in the manuscript:	_____
	Registration #	_____
	Registration date	_____
	Registry name and URL	_____
	Date of first patient randomized (verify that date of RCT registration is before the first patient enrolment in the study)	_____
Ethics	The ethics approval details appear in the manuscript:	_____
	Ethics committee/IRB approval reference #	_____
	Ethics committee/IRB approval date	_____
	Ethics committee/IRB name	_____
	Statement that all participants in the RCT gave appropriate informed consent before enrolment	_____
Sample size	The published sample size is close (≥ 85%) to the intended sample size stated in the registry entry *(Check protocol in registry against sample size stated in manuscript)*	_____
	Estimated sample size is appropriately calculated *(e.g. power and type‐1 error stated; target effect size and assumed rate in control group stated; standard deviation; any other adjustments such as the dropout or loss to follow‐up rates, justified. Post‐hoc analysis shows adequate sample size achieved. For pilot RCTs, sample size calculation is not needed.)*	_____
Author group		_____
Number	The RCT has at least three authors	_____
Methodology		_____
Selection bias	The allocation concealment is plausible and well explained *(e.g. two interventions but only one placebo, i.e. not matching for both interventions)*	_____
Plausibility	The methodology is reasonable for the type of intervention *(e.g. blinding of participants when this is not possible, as in surgeries or invasive procedures)*	_____
Randomization	Randomization method is clearly explained *(e.g. computer‐generated; block randomization, opaque envelopes; etc.)*	_____
Timeframe		_____
Recruitment	The number of actual recruited participants is reasonable in relation to eligible participants within the timeframe *(e.g. ideally RCT mentions total # deliveries in study centers during the study period; # eligible patients; # screened patients)*	_____
Submission	The manuscript was submitted at least 3 months after the last patient completed follow‐up *(Based on manuscript submission date, last randomization date and follow‐up date)*	_____
Dropout		_____
Withdrawals and lost‐to‐follow‐up	The authors address lost‐to‐follow‐up participants, and there are some patients lost to follow‐up *(Check in CONSORT algorithm; lost‐to‐follow‐up does not apply when the study is assessing an immediate outcome, e.g. a surgical outcome)*	_____
Rounded numbers	The dropout rate varies between groups, and the numbers do not appear to be artificially rounded *(e.g. groups of 50 or 100; mostly odd or even numbers, rounded to 10, etc.)*	_____
Baseline characteristics		_____
Number	At least five baseline characteristics are mentioned	_____
Homogeneity	Baseline characteristics do not appear perfectly balanced/do not seem implausible *(e.g. similar standard deviations for completely different characteristics with different means and distributions)*	_____
Omission	Important prognostic factors are mentioned as baseline characteristics	_____
Outcomes		_____
Effect size	The effect size is close to or similar to prior evidence *(e.g. if this is the only RCT showing positive results on this issue, the answer here should be ‘no’)*	_____
Conflicting information	The information provided is in agreement with the outcomes *(e.g. more ongoing pregnancies than clinical pregnancies in ART studies)*	_____
Author group		_____
Concerns	The authors do not have a history of previous manuscripts with retractions or EoCs (excluding papers withdrawn by the authors for valid – not trustworthiness‐related – reasons) If previous retractions or EoCs exist, consideration should be given to examining these authors' RCTs more closely *(Check each author on PubMed* ^7^ *and RetractionWatch* ^8^ *)*	_____

In general, if all criteria are not met, this may suggest lower quality of RCTs.

*A risk‐of‐bias assessment with tools (e.g. Cochrane) may include assessment of allocation concealment, randomization process, etc.

These do not have to be entered twice if already entered in risk‐of‐bias assessment tool.

The consensus decision was that the abstract and primary analysis of meta‐analyses should report only trustworthy, ‘high‐quality’ RCTs that meet all of the ‘absolute’ criteria (Table 1). Authors of meta‐analyses are encouraged to contact RCT authors for additional information regarding the criteria in Tables 1 and 2 if the details cannot be found in the published manuscript or registered protocol. At a minimum, all coauthors of meta‐analyses should confirm at the point of submission that each included article meets the criteria included in Table 1. Individual journals may also ask authors to confirm that each article meets the criteria in Table 2, or may go further and ask authors to complete and submit a checklist for the criteria in Tables 1 and 2 for each article included in the meta‐analysis. In general, RCTs that are published as an abstract only seldom report all criteria in Tables 1 and 2, and so would often not be included in meta‐analyses. Authors could consider a secondary analysis excluding RCTs that, while meeting all ‘absolute’ criteria, do not meet some of the ‘other quality’ criteria. Authors of meta‐analyses should also provide all items noted in the Submission Checklist for Meta‐analyses of RCTs (Table 3). A risk‐of‐bias assessment with tools (e.g. Cochrane) may include assessment of allocation concealment, randomization process, etc. These do not have to be entered twice (e.g. in Tables 1 and 2) if already entered in a risk‐of‐bias assessment tool.

**Table 3:** Submission checklist for meta‐analyses of RCTs

Checklist item	Completed/Reported ✓
All items in Tables 1 and 2 have been assessed and fulfilled	_______________
PROSPERO details	_______________
PRISMA checklist	_______________
Risk‐of‐bias assessment (e.g. Cochrane tools, etc.)	_______________

## DISCUSSION

Confirming trustworthiness of RCTs is crucial to the integrity of meta‐analyses, and assessment must begin with a thorough examination of the published RCTs being considered for inclusion. RCTs are more rigorous than other types of investigations, precisely because they are controlled. A meta‐analysis of RCTs combines those studies and extracts data to produce a pooled estimate, and tests for statistical significance. It is more robust than narrative reviews, and synthesizes the evidence rather than simply providing a review of the literature. A meta‐analysis of RCTs should meet the Cochrane criteria adopted by PRISMA (Preferred Reporting Items for Systematic Reviews and Meta‐Analyses), and include the necessary risk‐of‐bias assessment^6^. Subgroup or sensitivity analysis should be considered when there is a high variability or heterogeneity among studies.

A meta‐analysis is useful because it may overturn or amplify results from smaller RCTs. Preregistration is an important aspect in reducing bias, ensuring that the study protocols are registered prospectively (i.e. before the first participant is enrolled); changes to this plan during the course of the study can introduce bias. The International Committee of Medical Journal Editors (ICMJE) recommends mandatory prospective registration. Preregistration contributes to improving reproducibility of research, prevents duplication of efforts, and reduces the potential for bias. The most commonly used registry is ClinicalTrials.gov. Others are listed in the WHO Registry Network. The registry requires data on 24 mandatory elements of a study when preregistering. The study should follow CONSORT guidelines and the authors should have agreed to share the data if requested.

Retracted RCTs or low‐quality studies have often been included in meta‐analyses, including those with fabricated or plagiarized data. Evaluating RCTs against the ‘governance’ criteria outlined in Table 1 will help to address this issue. Including a risk‐of‐bias assessment of the included studies is important to evaluate the quality of the studies. Trustworthy meta‐analyses describe clearly the methods, search strategy, inclusion and exclusion criteria, data extraction procedures, and statistical analysis. If trustworthiness issues are raised, the RCT data should be shared upon request where available.

Including low‐quality articles (Table 2) in a meta‐analysis reduces the trustworthiness of the results. Trustworthy meta‐analyses mitigate publication bias through techniques such as funnel plot asymmetry, statistical tests and sensitivity analyses. Factors that contribute to trustworthiness include transparency, allowing evaluation of the thoroughness of the meta‐analysis.

Using the criteria suggested in this paper (Tables 1, 2, 3), authors can conduct trustworthy meta‐analyses that will promote the progression of science with integrity and reliability. The results may then be applied with confidence into standard clinical practice or clinical guidance.

## References

[1] Berghella V , Aviram A , Chescheir N , et al. Improving trustworthiness in research in women's health: a collective effort by OBGYN editors. Eur J Obstet Gynecol Reprod Biol. 2024;292:71‐74. 37976768 10.1016/j.ejogrb.2023.11.001

[2] Anderson K , Romero R , Odibo AO , et al. Quality criteria for randomized controlled studies: obstetrical journal guidelines. Am J Obstet Gynecol MFM. 2021;3:100334. 33607321 10.1016/j.ajogmf.2021.100334 PMC8324065

[3] Cochrane . Cochrane handbook for systematic reviews of interventions. Accessed May 17, 2024. https://training.cochrane.org/handbook/current.

[4] Mol BW , Lai S , Rahim A , et al. Checklist to assess Trustworthiness in RAndomised Controlled Trials (TRACT checklist): concept proposal and pilot. Res Integr Peer Rev. 2023;8(1):6. 37337220 10.1186/s41073-023-00130-8 PMC10280869

[5] Núñez‐Núñez M , Cano‐Ibáñez N , Zamora J , Bueno‐Cavanillas A , Khan KS . Assessing the integrity of clinical trials included in evidence syntheses. Int J Environ Res Public Health. 2023;20:6138. 37372725 10.3390/ijerph20126138 PMC10298200

[6] PRISMA . PRISMA 2020 checklist. Accessed May 17, 2024. https://www.prisma‐statement.org/prisma‐2020‐checklist.

[7] National Library of Medicine . PubMed Database. Accessed May 17, 2024. https://pubmed.ncbi.nlm.nih.gov/.

[8] RetractionWatch . The RetractionWatch Database. Accessed May 17, 2024. http://retractiondatabase.org/RetractionSearch.aspx.

[9] EQUATOR Network . CONSORT Checklist 2010. Accessed May 17, 2024. https://www.equator‐network.org/reporting‐guidelines/consort/.

